# Lipid profiling dataset of the Wnt3a-induced optic nerve regeneration

**DOI:** 10.1016/j.dib.2019.103966

**Published:** 2019-05-24

**Authors:** Anna Trzeciecka, Tal Carmy, Abigail S. Hackam, Sanjoy K. Bhattacharya

**Affiliations:** Bascom Palmer Eye Institute, Miller School of Medicine at University of Miami, Miami, FL, 33136, USA

**Keywords:** Optic nerve, Retina, Optic nerve crush, Regeneration, Wnt3a, Lipid profile

## Abstract

We present lipid profiling data from mouse retina and optic nerve after optic nerve crush and during Wnt3a-induced axonal regeneration at 7 and 15 days post-crush. This data is available at the Metabolomics Workbench, http://www.metabolomicsworkbench.org (Project ID: PR000718).

**Table:** Specifications table

Subject area	Biology
More specific subject area	Lipids
Type of data	Chromatograms, spectra, tables
How data was acquired	LC-MS/MS
Data format	Raw, filtered, analyzed
Experimental factors	Intact control, optic nerve crush + saline, optic nerve crush + Wnt3a
Experimental features	Mouse optic nerves and retinas were collected 7 and 15 days post-crush. After lipid extraction, samples were analysed using untargeted LC-MS/MS.
Data source location	Bascom Palmer Eye Institute, Miller School of Medicine at University of Miami, Miami, FL 33136, USA
Data accessibility	The Metabolomics Workbench (http://www.metabolomicsworkbench.org) - PR000718, https://doi.org/10.21228/M8VT2P
Related research article	[1]

**Table:** 

**Value of the Data** • Dataset explores retina and optic nerve lipid profiles after optic nerve crush and during Wnt3a-promoted axonal regeneration. • This data adds to the repertoire of axonal regeneration omics profiling datasets from different animal models and may be useful for integrative analysis and interpretation. • This dataset will be useful for the community of researchers studying optic neuropathies and optic nerve regeneration.

## 1 Data

**Fig. 1:**
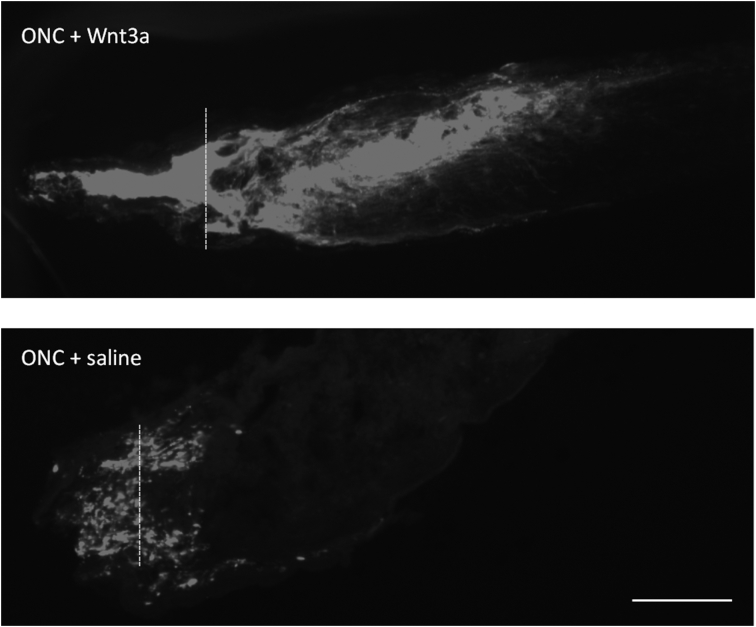
**Representative image of Wnt3a-induced axonal regeneration after optic nerve crush (ONC**). Regenerating axons (white) are visualized by retrograde labeling with Alexafluor 546 -conjugated cholera toxin B (CTB), the control was injected with phosphate buffered saline (PBS indicated as saline). The injury site is indicated by the dashed line. Minimal axonal outgrowth is observed in the saline injected injured nerve (ONC + saline), whereas robust regeneration is observed after Wnt3a injection (ONC + Wnt3a). Bar = 100 μm.

**Fig. 2:**
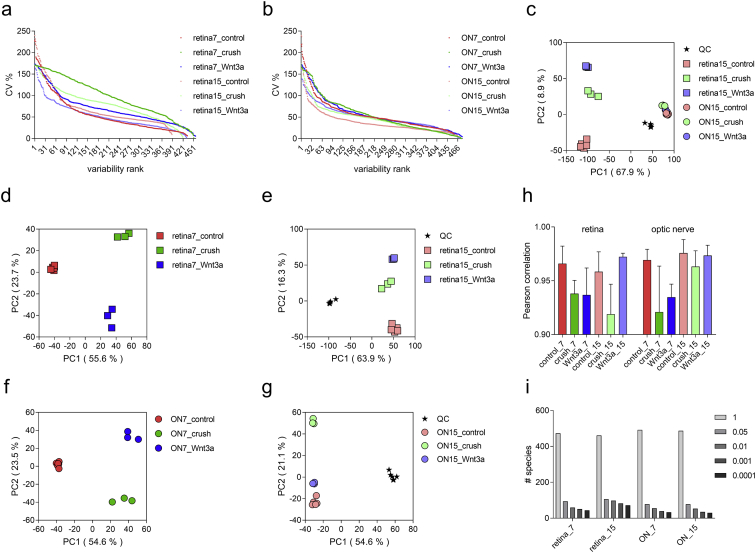
**Dataset overview**. (**a**-**b**) Relative variability (CV %) distribution among treatment groups: retina (**a**) and optic nerve (ON; **b**). (**c**-**g**) Principal component analysis (PCA) with samples plotted against their projections onto PC1 and PC2 (in brackets % fraction of variance explained by a principal component). QC is a pooled quality control sample that was run 6 times throughout 15 days post-crush analysis. (**h**) Pearson's correlation coefficients between biological replicates. Mean +SD. (**i**) Number of significant species at different FDR-adjusted p-value cut-offs for one-way ANOVA analysis.

**Fig. 3:**
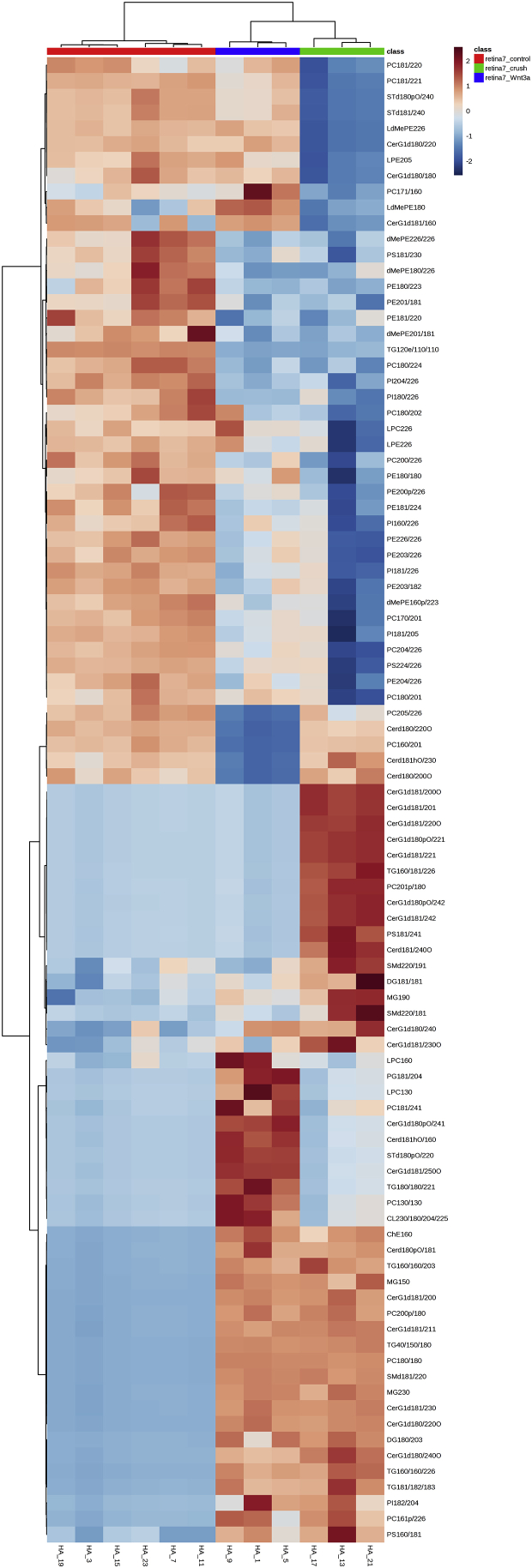
**Heatmap of the lipid abundance changes in retina 7 days post-crush.** 94 significant species are presented (FDR-adjusted p-value 0.05; one-way ANOVA). Ward clustering algorithm, Euclidean distance measure, autoscale features.

**Fig. 4:**
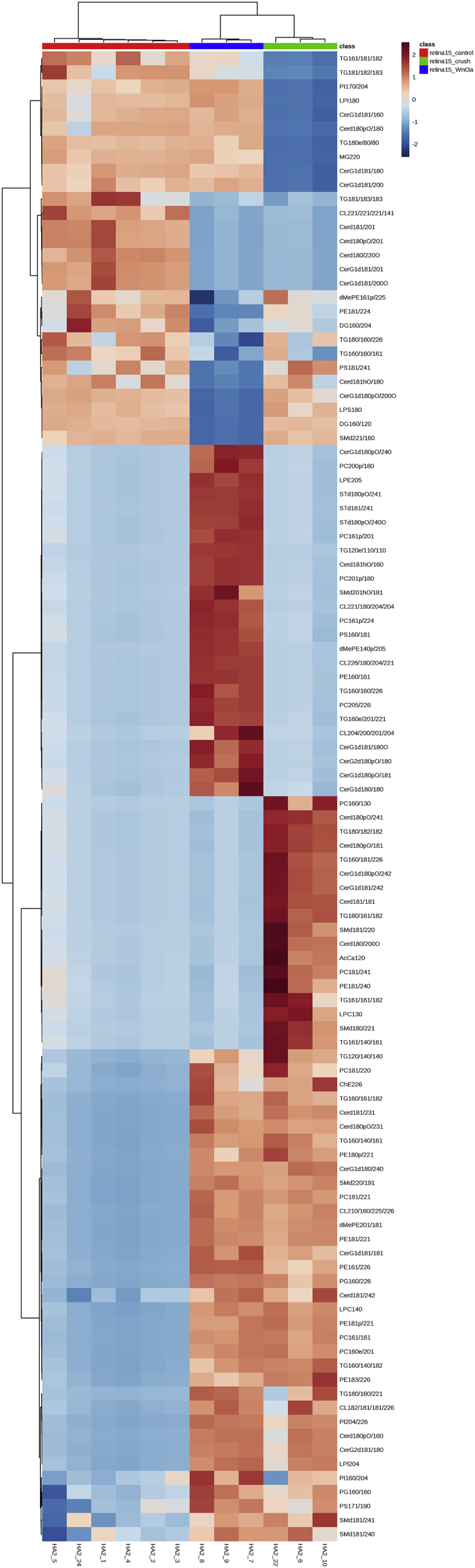
**Heatmap of the lipid abundance changes in retina 15 days post-crush.** 106 significant species are presented (FDR-adjusted p-value 0.05; one-way ANOVA). Ward clustering algorithm, Euclidean distance measure, autoscale features.

**Fig. 5:**
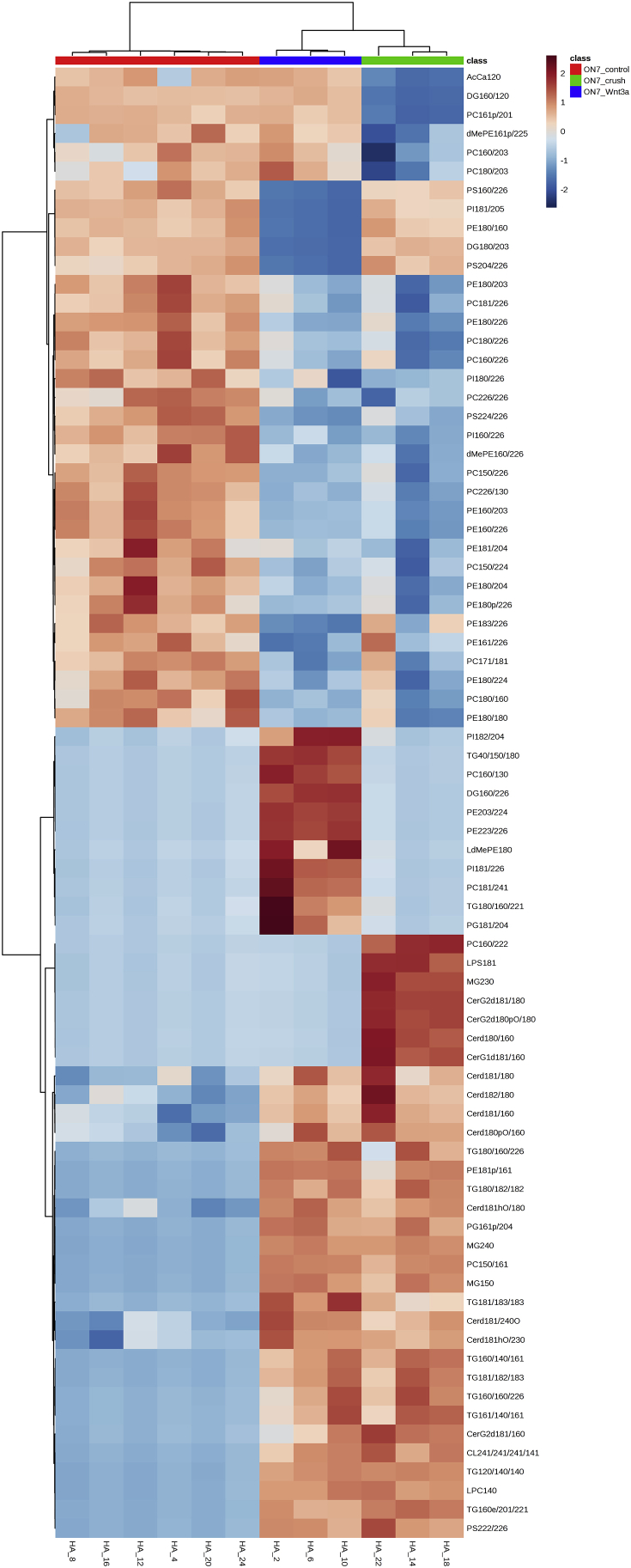
**Heatmap of the lipid abundance changes in optic nerve (ON) 7 days post-crush.** 78 significant species are presented (FDR-adjusted p-value 0.05; one-way ANOVA). Ward clustering algorithm, Euclidean distance measure, autoscale features.

**Fig. 6:**
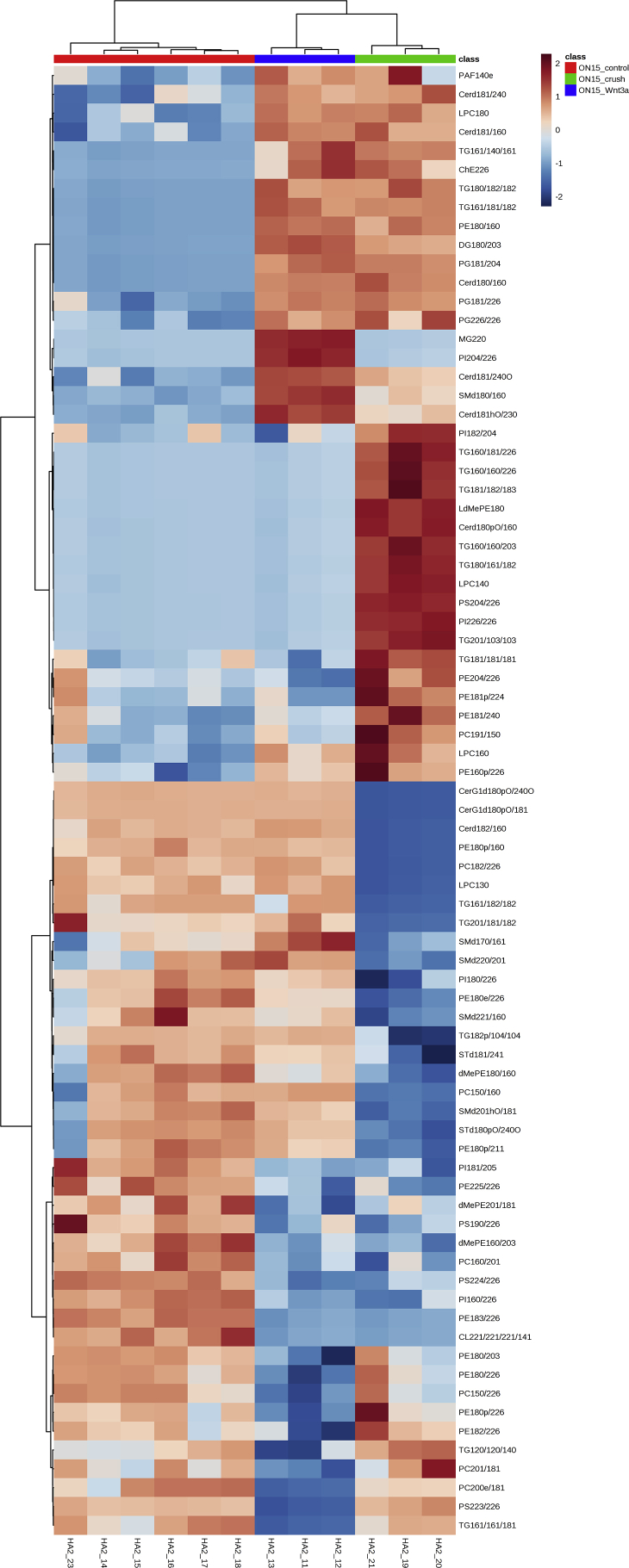
**Heatmap of the lipid abundance changes in optic nerve (ON) 15 days post-crush.** 78 significant species are presented (FDR-adjusted p-value 0.05; one-way ANOVA). Ward clustering algorithm, Euclidean distance measure, autoscale features.

We performed lipid profiling of the mouse retina and optic nerve after optic nerve crush followed by a single injection of saline (vehicle) or Wnt3a to induce retinal ganglion cells regeneration (Fig. 1). Specimens were collected 7 and 15 days post-crush, followed by chloroform-methanol based lipid extraction. Extracted lipid samples were analyzed using reversed-phase high-performance liquid chromatography (HPLC) with C30 column coupled to a Q Exactive mass spectrometer. Lipid identification and relative quantification were performed in LipidSearch software. Distributions of the CV % values for the experimental groups including intact controls are presented in Fig. 2 a-b. Each time-point groups of samples were clearly distinguished from quality control (QC) group and from each other with 75.7–80.2% of variance accounted for by PC1 and PC2 (Fig. 2 c-g). Biological replicates showed Pearson correlation coefficients ranging from 0.92 to 0.98 (Fig. 2 h). To identify features undergoing significant changes between experimental groups, we used one-way ANOVA analysis with Tukey's post-hoc test. We examined a number of significant features at different FDR adjusted p values for lipid species (Fig. 2 i). Next, we performed hierarchical clustering and heatmap visualization of the dysregulated species (FDR adjusted p values < 0.05). The heatmaps of significant species in the post-crush 7-day retina, 15-day retina, 7-day ON and 15-day ON are presented in Fig. 3, Fig. 4, Fig. 5, Fig. 6 respectively.

## 2 Experimental design, materials, and methods

### 2.1 ON crush and intravitreal injections

All procedures involving mice were performed in accordance with the ARVO Statement for the Use of Animals in Ophthalmic and Vision Research and were approved by the Animal Care and Use Committee at the University of Miami. The optic nerve crush and associated treatments were done on Tcf/LacZ mice. As described previously [1], the*Tcf/LacZ* mouse is a transgenic canonical Wnt reporter line that allows localization of active Wnt/β-catenin signaling [2]. In these mice, binding of endogenous nuclear β-catenin to T-cell factor/lymphoid enhancer-binding factor (TCF/Lef) elements in a Wnt-responsive enhancer/promoter region upstream of the *LacZ* transgene leads to induction of *LacZ*/β-galactosidase (β-gal) expression wherever Wnt signaling is active. The *Tcf/LacZ* strain was previously backcrossed onto the *C3H* background [3], and confirmed by PCR to lack the rd1 mutation.

Optic nerve crush (ONC) injury was performed as described previously [4]. A total of twelve Tcf/LacZ mice were used for this study. Six mice were used for a 7 day period and six mice were used for a 14 day period. The mice were used at the age of 6–8 weeks and were anesthetized using a ketamine/xylazine cocktail which was injected intraperitoneally. Once the mice were under the anesthesia, the eyes were locally anesthetized with 0.5% proparacaine hydrochloride. The mice were placed on a heating pad to begin the optic nerve crush procedure. Mice of either sex were randomly selected to receive intravitreal injection of recombinant 20ng Wnt3a ligand group or saline control group (saline was injected with equivalent volume as recombinant 20ng Wnt3a ligand). A small diabetic needle was used to make an incision in the superior posterior area of the conjunctiva-sclera border of the left eye. After which, either recombinant 20ng Wnt3a ligand or saline was injected intravitreally using a 1.5 cm 33-gauge Hamilton needle (Hamilton Company, Reno, NV). The injection needle was angled to avoid hitting the lens. The surface membrane of the left eye was cut around the conjunctiva-sclera border. Dumont #5 forceps were inserted between the membrane and the globe to move the surrounding tissues while searching for the optic nerve. Once the optic nerve was located, the forceps' teeth surrounded the nerve 1 mm from the globe and crushed the nerve for 5 seconds. The crush is successful if there is very minimal or no damage to the surrounding blood supply. Affected left eyes were treated with topical erythromycin ointment and 1mg/ml of Buprenorphine-SR lab was injected subcutaneously. Any mouse with excessive bleeding around the eye was excluded from the study. Mouse's overall health and left eye's health were monitored daily from day 1 post crush until day 7 post crush. Mice portraying lethargy 24 hours after surgery or eye infection to the affected eye days after surgery were excluded from the study. OCT scans were obtained from left and right eyes of the mouse while anesthetized on the day of euthanasia. Nerves and retinas were removed immediately after OCT scan for mass spectrometry analysis.

Regenerating RGC axons were anterogradely labeled using cholera toxin β subunit (CTB) with a conjugated Alexafluor 546 (Thermo Fisher Scientific, Waltham, MA) that was intravitreously injected in a volume of 2 μL at two days prior to animal euthanasia. The animals were then perfused using 4% paraformaldehyde and eyes and optic nerves were dissected and processed through a 5–20% sucrose gradient and then embedded in OCT Compound (Tissue Tek) for cyrosectioning. Longitudinal optic nerve sections were cut at a thickness of 8 μm and mounted onto slides and then imaged using a fluorescent microscope (Zeiss).

### 2.2 Lipid profiling

#### 2.2.1 Sample preparation

4 mL of methanol (LC-MS grade) and 2 mL of chloroform (LC-MS grade) were added to each sample. After 2 min vigorous vortexing and 2 min sonication in ultrasonic bath, the samples were incubated at 48 °C overnight (in borosilicate glass vials, PTFE-lined caps). The following day, 2 mL of water (LC-MS grade) and 1 mL of chloroform were added, samples vigorously vortexed for 2 min and centrifuged at 3000 RCF, 4 °C for 15 min to obtain phase separation. Lower phases were collected and dried in a centrifugal vacuum concentrator. Samples were stored at −20 °C until reconstituted in 150 μL of chloroform:methanol (1:1) prior to mass spectrometric analysis. Samples list is in Table 1. Lipid profiling was performed in 2 batches: 7 day and 15 day period. For the 15 day period, quality control (QC) pooled sample was prepared and run 6 times throughout the batch.

**Table 1:** Samples list.

Subject	Eye	Tissue	Day	ON crush	IVI injection		Lipid extraction protocol	LC-MS protocol	ESI mode	NCE	Data
					saline	20 ng of Wnt3a					
20F3J-1	OS	retina	7	+	–	+	+	+	+	15/30/45/60/75/90	HA_1_1-6
20F3J-1	OS	retina	7	+	–	+	+	+	–	15/30/45/60/75/90	HA_1_7-12
20F3J-1	OS	ON	7	+	–	+	+	+	+	15/30/45/60/75/90	HA_2_1-6
20F3J-1	OS	ON	7	+	–	+	+	+	–	15/30/45/60/75/90	HA_2_7-12
20F3J-1	OD	retina	7	–	–	–	+	+	+	15/30/45/60/75/90	HA_3_1-6
20F3J-1	OD	retina	7	–	–	–	+	+	–	15/30/45/60/75/90	HA_3_7-12
20F3J-1	OD	ON	7	–	–	–	+	+	+	15/30/45/60/75/90	HA_4_1-6
20F3J-1	OD	ON	7	–	–	–	+	+	–	15/30/45/60/75/90	HA_4_7-12
20F3J-2	OS	retina	7	+	–	+	+	+	+	15/30/45/60/75/90	HA_5_1-6
20F3J-2	OS	retina	7	+	–	+	+	+	–	15/30/45/60/75/90	HA_5_7-12
20F3J-2	OS	ON	7	+	–	+	+	+	+	15/30/45/60/75/90	HA_6_1-6
20F3J-2	OS	ON	7	+	–	+	+	+	–	15/30/45/60/75/90	HA_6_7-12
20F3J-2	OD	retina	7	–	–	–	+	+	+	15/30/45/60/75/90	HA_7_1-6
20F3J-2	OD	retina	7	–	–	–	+	+	–	15/30/45/60/75/90	HA_7_7-12
20F3J-2	OD	ON	7	–	–	–	+	+	+	15/30/45/60/75/90	HA_8_1-6
20F3J-2	OD	ON	7	–	–	–	+	+	–	15/30/45/60/75/90	HA_8_7-12
20F3J-3	OS	retina	7	+	–	+	+	+	+	15/30/45/60/75/90	HA_9_1-6
20F3J-3	OS	retina	7	+	–	+	+	+	–	15/30/45/60/75/90	HA_9_7-12
20F3J-3	OS	ON	7	+	–	+	+	+	+	15/30/45/60/75/90	HA_10_1-6
20F3J-3	OS	ON	7	+	–	+	+	+	–	15/30/45/60/75/90	HA_10_7-12
20F3J-3	OD	retina	7	–	–	–	+	+	+	15/30/45/60/75/90	HA_11_1-6
20F3J-3	OD	retina	7	–	–	–	+	+	–	15/30/45/60/75/90	HA_11_7-12
20F3J-3	OD	ON	7	–	–	–	+	+	+	15/30/45/60/75/90	HA_12_1-6
20F3J-3	OD	ON	7	–	–	–	+	+	–	15/30/45/60/75/90	HA_12_7-12
20F3J-4	OS	retina	7	+	+	–	+	+	+	15/30/45/60/75/90	HA_13_1-6
20F3J-4	OS	retina	7	+	+	–	+	+	–	15/30/45/60/75/90	HA_13_7-12
20F3J-4	OS	ON	7	+	+	–	+	+	+	15/30/45/60/75/90	HA_14_1-6
20F3J-4	OS	ON	7	+	+	–	+	+	–	15/30/45/60/75/90	HA_14_7-12
20F3J-4	OD	retina	7	–	–	–	+	+	+	15/30/45/60/75/90	HA_15_1-6
20F3J-4	OD	retina	7	–	–	–	+	+	–	15/30/45/60/75/90	HA_15_7-12
20F3J-4	OD	ON	7	–	–	–	+	+	+	15/30/45/60/75/90	HA_16_1-6
20F3J-4	OD	ON	7	–	–	–	+	+	–	15/30/45/60/75/90	HA_16_7-12
20F3J-5	OS	retina	7	+	+	–	+	+	+	15/30/45/60/75/90	HA_17_1-6
20F3J-5	OS	retina	7	+	+	–	+	+	–	15/30/45/60/75/90	HA_17_7-12
20F3J-5	OS	ON	7	+	+	–	+	+	+	15/30/45/60/75/90	HA_18_1-6
20F3J-5	OS	ON	7	+	+	–	+	+	–	15/30/45/60/75/90	HA_18_7-12
20F3J-5	OD	retina	7	–	–	–	+	+	+	15/30/45/60/75/90	HA_19_1-6
20F3J-5	OD	retina	7	–	–	–	+	+	–	15/30/45/60/75/90	HA_19_7-12
20F3J-5	OD	ON	7	–	–	–	+	+	+	15/30/45/60/75/90	HA_20_1-6
20F3J-5	OD	ON	7	–	–	–	+	+	–	15/30/45/60/75/90	HA_20_7-12
20F3I-2	OS	retina	7	+	+	–	+	+	+	15/30/45/60/75/90	HA_21_1-6
20F3I-2	OS	retina	7	+	+	–	+	+	–	15/30/45/60/75/90	HA_21_7-12
20F3I-2	OS	ON	7	+	+	–	+	+	+	15/30/45/60/75/90	HA_22_1-6
20F3I-2	OS	ON	7	+	+	–	+	+	–	15/30/45/60/75/90	HA_22_7-12
20F3I-2	OD	retina	7	–	–	–	+	+	+	15/30/45/60/75/90	HA_23_1-6
20F3I-2	OD	retina	7	–	–	–	+	+	–	15/30/45/60/75/90	HA_23_7-12
20F3I-2	OD	ON	7	–	–	–	+	+	+	15/30/45/60/75/90	HA_24_1-6
20F3I-2	OD	ON	7	–	–	–	+	+	–	15/30/45/60/75/90	HA_24_7-12
20F4D-6	OD	retina	15	–	–	–	+	+	+	15/30/45/60/75/90	HA2_1_1-6
20F4D-6	OD	retina	15	–	–	–	+	+	–	15/30/45/60/75/90	HA2_1_7-12
20F4D-5	OD	retina	15	–	–	–	+	+	+	15/30/45/60/75/90	HA2_2_1-6
20F4D-5	OD	retina	15	–	–	–	+	+	–	15/30/45/60/75/90	HA2_2_7-12
20F4D-2	OD	retina	15	–	–	–	+	+	+	15/30/45/60/75/90	HA2_3_1-6
20F4D-2	OD	retina	15	–	–	–	+	+	–	15/30/45/60/75/90	HA2_3_7-12
20F4D-1	OD	retina	15	–	–	–	+	+	+	15/30/45/60/75/90	HA2_4_1-6
20F4D-1	OD	retina	15	–	–	–	+	+	–	15/30/45/60/75/90	HA2_4_7-12
20F4D-3	OD	retina	15	–	–	–	+	+	+	15/30/45/60/75/90	HA2_5_1-6
20F4D-3	OD	retina	15	–	–	–	+	+	–	15/30/45/60/75/90	HA2_5_7-12
20F4D-5	OS	retina	15	+	+	–	+	+	+	15/30/45/60/75/90	HA2_6_1-6
20F4D-5	OS	retina	15	+	+	–	+	+	–	15/30/45/60/75/90	HA2_6_7-12
20F4D-2	OS	retina	15	+	–	+	+	+	+	15/30/45/60/75/90	HA2_7_1-6
20F4D-2	OS	retina	15	+	–	+	+	+	–	15/30/45/60/75/90	HA2_7_7-12
20F4D-1	OS	retina	15	+	–	+	+	+	+	15/30/45/60/75/90	HA2_8_1-6
20F4D-1	OS	retina	15	+	–	+	+	+	–	15/30/45/60/75/90	HA2_8_7-12
20F4D-6	OS	retina	15	+	–	+	+	+	+	15/30/45/60/75/90	HA2_9_1-6
20F4D-6	OS	retina	15	+	–	+	+	+	–	15/30/45/60/75/90	HA2_9_7-12
20F4D-3	OS	retina	15	+	+	–	+	+	+	15/30/45/60/75/90	HA2_10_1-6
20F4D-3	OS	retina	15	+	+	–	+	+	–	15/30/45/60/75/90	HA2_10_7-12
20F4D-2	OS	ON	15	+	–	+	+	+	+	15/30/45/60/75/90	HA2_11_1-6
20F4D-2	OS	ON	15	+	–	+	+	+	–	15/30/45/60/75/90	HA2_11_7-12
20F4D-6	OS	ON	15	+	–	+	+	+	+	15/30/45/60/75/90	HA2_12_1-6
20F4D-6	OS	ON	15	+	–	+	+	+	–	15/30/45/60/75/90	HA2_12_7-12
20F4D-1	OS	ON	15	+	–	+	+	+	+	15/30/45/60/75/90	HA2_13_1-6
20F4D-1	OS	ON	15	+	–	+	+	+	–	15/30/45/60/75/90	HA2_13_7-12
20F4D-1	OD	ON	15	–	–	–	+	+	+	15/30/45/60/75/90	HA2_14_1-6
20F4D-1	OD	ON	15	–	–	–	+	+	–	15/30/45/60/75/90	HA2_14_7-12
20F4D-6	OD	ON	15	–	–	–	+	+	+	15/30/45/60/75/90	HA2_15_1-6
20F4D-6	OD	ON	15	–	–	–	+	+	–	15/30/45/60/75/90	HA2_15_7-12
20F4D-2	OD	ON	15	–	–	–	+	+	+	15/30/45/60/75/90	HA2_16_1-6
20F4D-2	OD	ON	15	–	–	–	+	+	–	15/30/45/60/75/90	HA2_16_7-12
20F4D-5	OD	ON	15	–	–	–	+	+	+	15/30/45/60/75/90	HA2_17_1-6
20F4D-5	OD	ON	15	–	–	–	+	+	–	15/30/45/60/75/90	HA2_17_7-12
20F4D-3	OD	ON	15	–	–	–	+	+	+	15/30/45/60/75/90	HA2_18_1-6
20F4D-3	OD	ON	15	–	–	–	+	+	–	15/30/45/60/75/90	HA2_18_7-12
20F4D-3	OS	ON	15	+	+	–	+	+	+	15/30/45/60/75/90	HA2_19_1-6
20F4D-3	OS	ON	15	+	+	–	+	+	–	15/30/45/60/75/90	HA2_19_7-12
20F4D-5	OS	ON	15	+	+	–	+	+	+	15/30/45/60/75/90	HA2_20_1-6
20F4D-5	OS	ON	15	+	+	–	+	+	–	15/30/45/60/75/90	HA2_20_7-12
20F4E-1	OS	ON	15	+	+	–	+	+	+	15/30/45/60/75/90	HA2_21_1-6
20F4E-1	OS	ON	15	+	+	–	+	+	–	15/30/45/60/75/90	HA2_21_7-12
20F4E-1	OS	retina	15	+	+	–	+	+	+	15/30/45/60/75/90	HA2_22_1-6
20F4E-1	OS	retina	15	+	+	–	+	+	–	15/30/45/60/75/90	HA2_22_7-12
20F4E-1	OD	ON	15	–	–	–	+	+	+	15/30/45/60/75/90	HA2_23_1-6
20F4E-1	OD	ON	15	–	–	–	+	+	–	15/30/45/60/75/90	HA2_23_7-12
20F4E-1	OD	retina	15	–	–	–	+	+	+	15/30/45/60/75/90	HA2_24_1-6
20F4E-1	OD	retina	15	–	–	–	+	+	–	15/30/45/60/75/90	HA2_24_7-12
**Quality control (QC) sample pooled from HA2_1-24**											
N/A	N/A	N/A	N/A	N/A	N/A	N/A	N/A	+	+	15/30/45/60/75/90	QC1_1-6
N/A	N/A	N/A	N/A	N/A	N/A	N/A	N/A	+	–	15/30/45/60/75/90	QC1_7-12
N/A	N/A	N/A	N/A	N/A	N/A	N/A	N/A	+	+	15/30/45/60/75/90	QC2_1-6
N/A	N/A	N/A	N/A	N/A	N/A	N/A	N/A	+	–	15/30/45/60/75/90	QC2_7-12
N/A	N/A	N/A	N/A	N/A	N/A	N/A	N/A	+	+	15/30/45/60/75/90	QC3_1-6
N/A	N/A	N/A	N/A	N/A	N/A	N/A	N/A	+	–	15/30/45/60/75/90	QC3_7-12
N/A	N/A	N/A	N/A	N/A	N/A	N/A	N/A	+	+	15/30/45/60/75/90	QC4_1-6
N/A	N/A	N/A	N/A	N/A	N/A	N/A	N/A	+	–	15/30/45/60/75/90	QC4_7-12
N/A	N/A	N/A	N/A	N/A	N/A	N/A	N/A	+	+	15/30/45/60/75/90	QC5_1-6
N/A	N/A	N/A	N/A	N/A	N/A	N/A	N/A	+	–	15/30/45/60/75/90	QC5_7-12
N/A	N/A	N/A	N/A	N/A	N/A	N/A	N/A	+	+	15/30/45/60/75/90	QC6_1-6
N/A	N/A	N/A	N/A	N/A	N/A	N/A	N/A	+	–	15/30/45/60/75/90	QC6_7-12

#### 2.2.2 High performance liquid chromatography (HPLC)

Reversed phase chromatographic separation utilized Accela Autosampler (Thermo), Accela 600 pump (Thermo) and Acclaim C30 column: 3 μm, 2.1 × 150 mm (Thermo). The column temperature was maintained at 30 °C and tray temperature at 20 °C. Solvent A was composed of 10 mM ammonium acetate (LC-MS grade) in 60:40 methanol:water (LC-MS grade) with 0.2% formic acid (FA; LC-MS grade). Solvent B was composed of 10 mM ammonium acetate with 60:40 methanol:chloroform with 0.2% FA. The flow rate was 260 μL/min and injection volume was 10 μL. The gradient was 35–100% solvent B over 13.0 min, 100% solvent B over 13.0–13.8 min, 100-35% solvent B over 13.8–14.5 min, 35% solvent B over 14.5–18.0 min, 0% solvent B over 18.0–20.0 min.

#### 2.2.3 Mass spectrometry

The Q Exactive (Thermo) mass spectrometer was operated under heated electrospray ionization (HESI) in positive and negative mode separately. The spray voltage was 4.4 kV, the heated capillary was held at 310 °C (negative mode) or 350 °C (positive mode) and heater at 275 °C (positive mode). The S-lens radio frequency (RF) level was 70. The sheath gas flow rate was 30 (negative mode) or 45 units (positive mode) and auxiliary gas was 14 (negative mode) or 15 units (positive mode). Full scan used resolution 70,000 with automatic gain control (AGC) target of 1 × 10^6^ ions and maximum ion injection time (IT) of 100 ms. Data-dependent MS/MS (top10) were acquired using the following parameters: resolution 17,500; AGC 1 × 10^5^; maximum IT 75 ms; 1.3 m/z isolation window; underfill ratio 0.1%; intensity threshold 1 × 10^3^; dynamic exclusion time 3 s. Normalized collision energy (NCE) settings were: 15, 30, 45, 60, 75, 90 (in positive and negative mode separately; total 12 runs per sample). Total instrument time ∼216 h. External mass calibration was performed using the standard calibration mixture. Each sample was preceded and followed by a blank run (1:1 chloroform:methanol).

#### 2.2.4 Lipid identification and quantification

Lipid identification and relative quantification were performed using LipidSearch 4.1 software (Thermo). The search criteria were as follows: product search; parent m/z tolerance 5 ppm; product m/z tolerance 5 ppm; quantification: m/z tolerance 5 ppm, retention time tolerance 1 min. The following adducts were allowed in positive mode: +H, +NH4, +H-H2O, +H-2H2O, +2H, +Na, +K and negative mode: -H, +HCOO, +CH3COO, -2H, -Cl. All classes were selected for search. LipidSearch nomenclature is used (Table 2).

**Table 2:** LipidSearch nomenclature.

Group	ClassKey	Lipid name
phospholipid	dMePE	dimethylphosphatidylethanolamine
	LdMePE	lysodimethylphosphatidylethanolamine
	LPC	lysophosphatidylcholine
	LPE	lysophosphatidylethanolamine
	LPI	lysophosphatidylinositol
	LPS	lysophosphatidylserine
	PA	phosphatidic acid
	PAF	platelet-activating factor
	PC	phosphatidylcholine
	PE	phosphatidylethanolamine
	PEt	phosphatidylethanol
	PG	phosphatidylglycerol
	PI	phosphatidylinositol
	PMe	phosphatidylmethanol
	PS	phosphatidylserine
sphingolipid	Cer	ceramide
	SM	sphingomyelin
	So	sphingosine
glycosphingolipid	CerG1	hexosyl ceramide
	CerG2	dihexosyl ceramide
	ST	sulfatide
cardiolipin	CL	cardiolipin
neutral glycerolipid	MG	monoglyceride
	DG	diglyceride
	TG	triglyceride
steroid	ChE	cholesterol ester
coenzyme	Co	coenzyme
fatty esters	AcCa	acyl carnitine

#### 2.2.5 Data processing

Positive and negative mode identifications at different NCE were aligned in LipidSearch, allowing calculation of unassigned peaks. The following settings were applied: product search; alignment method max; retention time tolerance 0.1 min; filters: toprank, main isomer peak; M-score 5; molecular lipid identification grade: A-B (A: lipid class and fatty acid completely identified or B: lipid class and some fatty acid identified). Peaks of the following lipid classes: BisMePA, SQMG, MGDG and MGMG, were considered false positives and were removed. Only peaks appearing in all replicates were accepted. Peaks with the same annotated lipid species were merged. Lists of species and their main areas were uploaded to Metaboanalyst 4.0 [5] statistical analysis module. Missing values were replaced with a small number (half of the minimum positive value in the original data). Normalization to median and log2 transformation were applied.
