# Supplementary material for: Lipid profiling dataset of the Wnt3a-induced optic nerve regeneration
Source: Data Brief. 2019 May 24;25:103966. doi: 10.1016/j.dib.2019.103966 (PMC6723204; doi:10.1016/j.dib.2019.103966)
Supplement: Multimedia component 1 [file mmc1.pdf]

Bascom Palmer Eye Institute
UNIVERSITY OF MIAMI HEALTH SYSTEM

To
The co-Editors-in-Chief/Managing Editor
Data-in-Brief

Conflict of Interest Statement

Manuscript No.: Manuscript No.: DIB-D-19-00054
Title: Lipid profiling dataset of the Wnt3a-induced optic nerve regeneration Journal Title: Data in Brief Corresponding Author: Professor Sanjoy K. Bhattacharya All Authors: Anna Trzeciecka, MD; Sanjoy K. Bhattacharya, Ph.D Submit Date: Jan 08, 2019

Dear Editor/Co-editors-in Chief,

On behalf of all authors, I declare that there is no conflict of interest whatsoever.

'Declarations of interest: none'.

Sincerely,

Sanjoy Bhattacharya, M. Tech., Ph. D.
Professor of Ophthalmology, Neuroscience, Biochemistry and Molecular Biology
Director, Miami Integrative Metabolomics Research Center (MIMRC)
